# Subclinical Anthracycline-Induced Cardiotoxicity in the Long - Term Follow-Up of Lymphoma Survivors: A Multi-Layer Speckle Tracking Analysis

**DOI:** 10.5935/abc.20180042

**Published:** 2018-03

**Authors:** Yu Kang, Fei Xiao, Haiyan Chen, Wei Wang, Lijing Shen, Hang Zhao, Xuedong Shen, Fangyuan Chen, Ben He

**Affiliations:** 1 Department of Cardiology - Renji Hospital - School of Medicine - Shanghai Jiaotong University, Shanghai - China; 2 Department of Hemotology - Renji Hospital - School of Medicine - Shanghai Jiaotong University, Shanghai - China; 3 Department of Echocardiography - Zhongshan Hospital of Fudan University, Shanghai - China

**Keywords:** Cardiotoxicity, Anthracyclines, Lymphoma, Non-Hodgkin, Hematologic Neoplasias/drug therapy, Echocardiography

## Abstract

**Background:**

Anthracycline generates progressive left ventricular dysfunction associated with a poor prognosis.

**Objectives:**

The purpose of this study was to evaluate whether layer-specific strain analysis could assess the subclinical left ventricular dysfunction after exposure to anthracycline.

**Methods:**

Forty-two anthracycline-treated survivors of large B-cell non-Hodgkin lymphoma, aged 55.83 ± 17.92 years (chemotherapy group) and 27 healthy volunteers, aged 51.39 ± 13.40 years (control group) were enrolled. The cumulative dose of epirubicin in chemotherapy group was 319.67 ± 71.71mg/m^2^. The time from last dose of epirubicin to the echocardiographic examination was 52.92 ± 22.32 months. Global longitudinal (GLS), circumferential (GCS) and radial strain (GRS), subendocardial, mid and subepicardial layer of longitudinal (LS-ENDO, LS-MID, LS-EPI) and circumferential strain (CS-ENDO, CS-MID, CS-EPI) values were analyzed. Transmural strain gradient was calculated as differences in peak systolic strain between the subendocardial and subepicardial layers. A value of p < 0.05 was considered significant.

**Results:**

Conventional parameters of systolic and diastolic function showed no significant difference between two groups. Compared with controls, patients had significantly lower GCS and GLS. Multi-layer speckle tracking analysis showed significant reduction of circumferential strain of subendocardial layer, transmural CS gradient and longitudinal strain of all three layers. In contrast, the two groups did not differ in transmural longitudinal strain gradient and radial strains.

**Conclusions:**

It proved the preferential impairment of subendocardial deformation in long-term survivors after exposure to anthracycline. Multi-layer speckle tracking echocardiography might facilitate the longitudinal follow-up of this at-risk patient cohort.

## Introduction

Anthracycline, a commonly used chemotherapeutic agent in the treatment of a wide spectrum of hematologic malignancies and solid tumors, is undermined by potential life-threatening cardiotoxicity.^1,2^ Anthracycline-induced left ventricular dysfunction is believed to be refractory to conventional pharmacological therapy and to be associated with a poor prognosis. Therefore, detection of subclinical myocardial dysfunction is of vital importance to balance between the cardiac risk and the potential cancer treatment.

Two-dimensional speckle tracking echocardiography, based on tracking local image details from frame to frame throughout the cardiac cycle,^3-6^ has allowed more accurate measurements of regional myocardial systolic performance.

It has been proved that patients treated with epirubicin-based chemotherapy experienced significant decrease in strain values while LVEF remained stable and within normal limits.^7,8^ Based on the 2D speckle tracking technology, a novel offline tool is recently introduced which has a potential of measuring strains in subendocardial and subepicardial layers comparatively.

Therefore, the objectives of this study were to evaluate whether layer-specified systolic strain analysis could differentiate the subclinical left ventricular function changes in patients after exposure to anthracycline-based chemotherapy.

## Methods

### Study population

A total of 45 anthracycline-treated survivors of histopathologically confirmed large B-cell non-Hodgkin lymphoma who have been off treatment for at least one year were enrolled in this study between March 2014 and Dec 2015 (chemotherapy group). Exclusion criteria were uncontrolled hypertension, significant valvular disease, congenital disease, a widened QRS complex on surface ECG, arrhythmia, a previous history of heart failure and/or coronary artery disease. The following data were collected including date of completion of chemotherapy, duration of follow-up, cumulative doses of anthracyclines, symptoms and signs of heart failure, and cardiac medications. Twenty-eigh age-matched and gender-matched referred to our hospital for non-specific chest pain or palpitation, but with no history of cardiovascular disease patients and with completely normal electrocardiograms, echocardiograms, treadmill stress exercises and 24-hour, continuous ambulatory electrocardiograms were selected as controls (control group).

### Echocardiographic imaging

Images were obtained in the left lateral decubitus position with Vivid E9 (GE Healthcare, Horton, Norway) ultrasound systems. Standard two-dimensional images were acquired according to recommendations of the American Society of Echocardiography.^9^ Depth was minimized to optimize the frame rate. At least three beats were digitally stored for offline analysis. Left ventricular ejection fraction was calculated using the modified Simpson's biplane method. Left ventricular mass index, relative wall thickness, transmitral peak early (E) and peak late (A) diastolic filling velocities were also measured. Tissue Doppler echocardiography was performed with the sample volume positioned at the basal LV free wall and septum at the mitral annular junction to obtain lateral and septal mitral annular systolic (S') and early diastolic myocardial tissue velocities (E').

### Multi-layer speckle tracking echocardiography

Gray scale images for offline speckle tracking analysis were acquired at frame rate of 53 to 84 MHz. Echopac version 11.1 (GE Healthcare, Horton, Norway) was used for multi-layer strain analysis. The automatic tracking analysis was performed in the apical 4-chamber, 2-chamber, long-axis apical view for longitudinal strain and in the parasternal short-axis view at basal, mid-papillary and apical level for circumferential and radial strain according to the vendor's instructions. The endocardial border was manually traced at end-diastole. The ROI (region of interest) for strain analysis was adjusted manually. The locations of the tracking points were adjusted when necessary so that the region of interest extended from endocardial to epicardial borders to approximate the myocardium, which was divided into subendocardial, middle and subepicardial myocardium layers of equal thickness.

Peak circumferential (CS) and radial strain (RS) measurements were obtained from the basal, mid-segments of the septal, lateral, inferior, anterior, anteroseptal, posterior walls, apical segments of anterior, inferior, septal, lateral walls, totally 18 segments. Peak longitudinal strain (LS) measurements were obtained from the basal, mid- and apical segments of the anterior, inferior, anteroseptal, anterolateral, inferoseptal, inferolateral walls, totally 16 segments. In each segment, the subendocardial, middle and subepicardial LS and CS were calculated automatically. Regional strain values were averaged to determine global longitudinal/circumferential/radial strain (GLS, GCS, GRS), global subendocardial, middle and subepicardial LS (LS-ENDO, LS-MID, LS-EPI) and CS (CS-ENDO, CS-MID, CS-EPI). Transmural strain gradient was calculated as differences of peak systolic strain between the subendocardial and subepicardial layers. Strain values of each level were calculated.

### Reproducibility

Intra- and inter-observer reproducibility was assessed by calculating the difference between the values of 10 randomly selected patients measured by one observer twice and by a second observer.

### Statistical analysis

Continuous variables with normal distribution were expressed as the mean ± standard deviation. Continuous variables with non-normal distribution were expressed as median and interquartile range. Differences between two groups were determined using independent samples t test for continuous variables with normal distribution and Kruskal Wallis test for with non-normal distribution. One sample K-S test was used in determining the normality of data. One way ANOVA test was used to compare the differences between strain values of different layers and different levels within each group. Relations between strain values and cumulative anthracycline dose were determined by Pearson correlation analysis. Interobserver and intraobserver reproducibility of strain values were assessed using intraclass correlation coefficients (ICCs) and Bland-Altman analysis. Data were analyzed by SPSS version 16.0 (SPSS, Inc, Chicago, IL, USA).

A value of p < 0.05 was considered significant.

## Results

Three patients and one healthy volunteer were excluded from the analysis because of poor image quality (defined as > 2 non-visualized segments). Forty-two patients, 18 males, ranging in age from 22 to 77 years (mean age 55.83 ± 17.92 years), and 27 healthy volunteers, 14 males, ranging in age from 32 to 77 years (mean age 51.39 ± 13.40 years), were finally included in the statistical analysis. Table 1 shows the two groups clinical characteristics. In all patients, the cumulative dose of epirubicin was 319.67 ± 71.71 mg/m^2^ (ranging from 150.94 mg/m^2^ to 440.00 mg/m^2^). Patients have not received radiotherapy or other cardiotoxic agents. No patient complained of cardiovascular related symptoms. EKG remained normal in all patients. The time from last dose of epirubicin to the echocardiographic examination was 52.92 ± 22.32 months (ranging from 24 months to 104 months).

**Table 1:** Clinical characteristics of two groups

	Normal	Chemotherapy	p value
Number	27	42	
Male (n/%)	12 (44.44)	18 (42.86)	0.84
Age (y)	50.39 ± 13.40	55.83 ± 17.92	0.16
Hypertension (n/%)	0(0)	4(9.52)	
ACEI (n/%)	0(0)	1(2.38)	
ARB (n/%)	0 (0)	1(2.38)	
CCB (n/%)	0(0)	0(0)	
β-blocker (n/%)	0(0)	1(2.38)	
Smoker (n/%)	5(17.24)	9(21.42)	0.470
DM (n/%)	0(0)	1(2.38)	
SBP (mmHg)	124.8 ± 12.6	121.6 ± 12.5	0.627
DBP (mmHg)	70.7 ± 9.3	69.5 ± 7.9	0.233
HR (bpm)	78.0 ± 11.3	81.0 ± 14.5	0.099

ACEI: angiotensin-converting enzyme inhibitors; ARB: angiotensin receptor blockers; CCB: calcium-channel blocker; DBP: diastolic blood pressure; DM: diabetes mellitus; HR: heart rate; SBP: systolic blood pressure; p values were assessed by independent samples t test

### Conventional echocardiographic parameters

Table 2 summarized the echocardiographic findings in the two groups. Conventional parameters of systolic and diastolic function, including body surface area indexed left ventricular end-diastolic volume (LVEDV/BSA), body surface area indexed left ventricular end-systolic volume (LVESV/BSA), left ventricular ejection fraction, E velocity, A velocity, E/A ratio, deceleration time, S' velocity, E/E', isovolumic relaxation time showed no significant difference between two groups.

**Table 2:** Conventional echocardiographic parameters between two groups.

	Normal	Chemotherapy	p value
LVEDV/BSA (ml)	47.22 ± 13.97	46.99 ± 13.99	0.95
LVESV/BSA (ml)	16.31 ± 6.24	16.30 ± 6.47	0.99
LVMI (g/m^2^)	79.32 ± 16.66	71.87 ± 13.68	0.13
RWT	0.36 ± 0.05	0.36 ± 0.05	0.93
LVEF (%)	66.46 ± 5.55	66.04 ± 6.52	0.78
E velocity (m/s)	80.38 ± 24.11	72.45 ± 16.99	0.11
A velocity (m/s)	76.62 ± 17.76	76.61 ± 19.07	0.95
E/A ratio	1.11 ± 0.44	1.01 ± 3.53	0.32
S' velocity (m/s)	9.50 ± 2.19	9.24 ± 2.08	0.60
E/E' ratio	6.95 ± 3.21	6.71 ± 2.31	0.71
DT (ms)	145.88 ± 27.81	149.95 ± 34.28	0.61
IVRT (ms)	85.36 ± 20.14	88.13 ± 24.77	0.62

BSA: body surface area; DT: deceleration time; FS: fraction shortening; LVEDV: left ventricular end-diastolic volume; LVEF: left ventricular ejection fraction; LVESV: left ventricular end-systolic volume; LVMI: left ventricular mass index; IVRT: isovolumic relaxation time; RWT: relative wall thickness; p values were assessed by independent samples t test

### Multi-layer speckle tracking echocardiography

**Figure 1:**
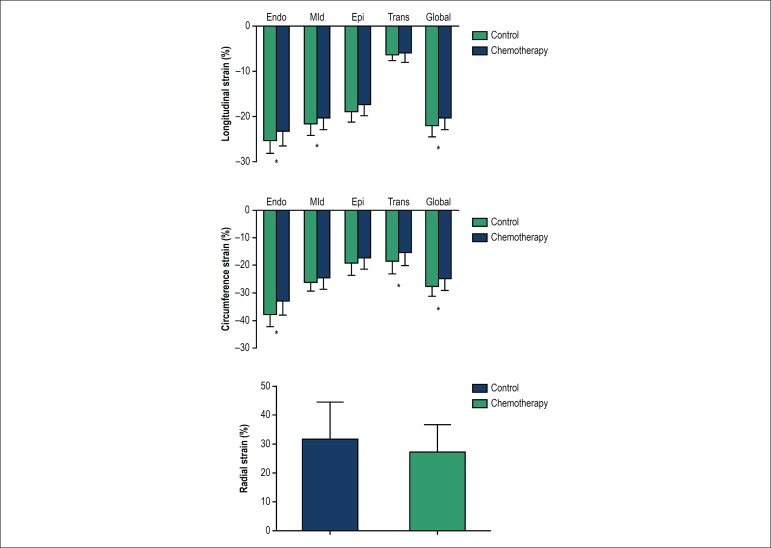
Strain values between two groups. *:p < 0.05.

In both groups, longitudinal and circumferential strains were highest in the apical region and decreased significantly from apical to basal level (Table 3,4). The left ventricular longitudinal and circumferential strains of different myocardial layers in patients and controls are shown in Table 5, Figure 1. Transmural strain gradients in LS and CS were demonstrated in both patients and controls, with strain values decreasing from the subendocardial to subepicardial layers. GCS was significantly decreased in chemotherapy group respect to control group (-27.73% ± 3.37% vs -24.94% ± 4.14%, p = 0.004). The reduction of GCS was attributable to significantly reduced CS-ENDO but preserved CS-EPI strain in patients compared with controls. The longitudinal strain values of global left ventricle and all three layers were significantly decreased in chemotherapy group. However, the two groups did not differ in transmural longitudinal strain gradient. In contrast, there was no statistic difference in radial strains between the two groups.

**Table 3:** Three-layer circumferential strain values between two groups stratified by levels

	CS-Endo(%)			CS-Mid(%)			CS-Epi(%)		
	Control	Chemotherapy	P value	Control	Chemotherapy	P value	Control	Chemotherapy	p value
Basal level	-33.48 ± 5.10	-26.26 ± 4.34	0.000	-23.95 ± 4.26	-22.37 ± 4.28	0.149	-17.58 ± 4.03	-16.85 ± 3.93	0.453
Mid level	-34.29 ± 4.21	-31.25 ± 5.39	0.014	-24.45 ± 3.46	-22.57 ± 3.67	0.053	-17.54 ± 3.21	-16.85 ± 3.94	0.063
Apical level	-44.31 ± 6.14	-41.13 ± 9.47	0.038	-30.32 ± 4.46	-28.91 ± 6.34	0.316	-21.77 ± 3.95	-19.81 ± 5.39	0.105
P value	0.000	0.000		0.000	0.000		0.000	0.000	

CS-ENDO: subendocardial circumferential strain; CS-EPI: subepicardial circumferential strain; CS-MID: middle circumferential strain. P values were analyzed by one way ANOVA test.

**Table 4:** Three-layer longitudinal strain values between two groups stratified by levels

	LS-Endo(%)			LS-Mid(%)			LS-Epi(%)		
	Control	Chemotherapy	P value	Control	Chemotherapy	P value	Control	Chemotherapy	p value
Basal level	-18.51 ± 2.55	-16.82 ± 2.36	0.006	-17.74 ± 2.50	-16.49 ± 2.11	0.027	-17.07 ± 2.50	-15.63 ± 2.00	0.009
Mid level	-22.76 ± 2.72	-21.04 ± 2.87	0.014	-20.82 ± 2.39	-19.45 ± 2.49	0.028	-19.23 ± 2.56	-16.87 ± 2.26	0.000
Apical level	-34.36 ± 3.23	-32.29 ± 5.69	0.101	-26.20 ± 3.06	-25.08 ± 4.23	0.623	-20.8 ± 2.55	-19.53 ± 5.12	0.538
P value	0.000	0.000		0.000	0.000		0.000	0.000	

LS-ENDO: subendocardial longitudinal strain; LS-EPI: subepicardial longitudinal strain; LS-MID: middle longitudinal strain. P values were analyzed by one way ANOVA test.

**Table 5:** Strain values between two groups.

	Control	Chemotherapy	p value
GLS (%)	-21.86 ± 2.38	-20.36 ± 2.58	0.016*
GCS (%)	-27.73 ± 3.37	-24.94 ± 4.14	0.004*
GRS (%)	31.44 ± 12.98	26.89 ± 9.75	0.118
LS-ENDO(%)	-25.21 ± 2.72	-23.38 ± 3.11	0.014*
LS-MID(%)	-21.53 ± 2.36	-20.35 ± 2.58	0.029*
LS-EPI(%)	-18.83 ± 2.19	-17.35 ± 2.48	0.013*
LS gradient (%)	-6.38 ± 1.28	-6.03 ± 2.07	0.439
CS-ENDO(%)	-37.37 ± 3.79	-32.88 ± 5.23	0.000*
CS-MID(%)	-26.24 ± 2.98	-24.62 ± 4.13	0.073
CS-EPI(%)	-19.56 ± 4.45	-17.32 ± 4.13	0.066
CS gradient(%)	-17.80 ± 3.69	-15.55 ± 4.59	0.0*

CS-ENDO: subendocardial circumferential strain; CS-EPI: subepicardial circumferential strain; CS-MID: middle circumferential strain; GCS: global circumferential strain; GLS: global longitudinal strain; GRS: global radial strain; LS-ENDO: subendocardial longitudinal strain; LS-EPI: subepicardial longitudinal strain; LS-MID: middle longitudinal strain.

* p < 0.05. p values were assessed by independent samples t test

There was no correlation between anthracycline dose and layer specific strain values.

### Inter and intra-observer variation

**Figure 2:**
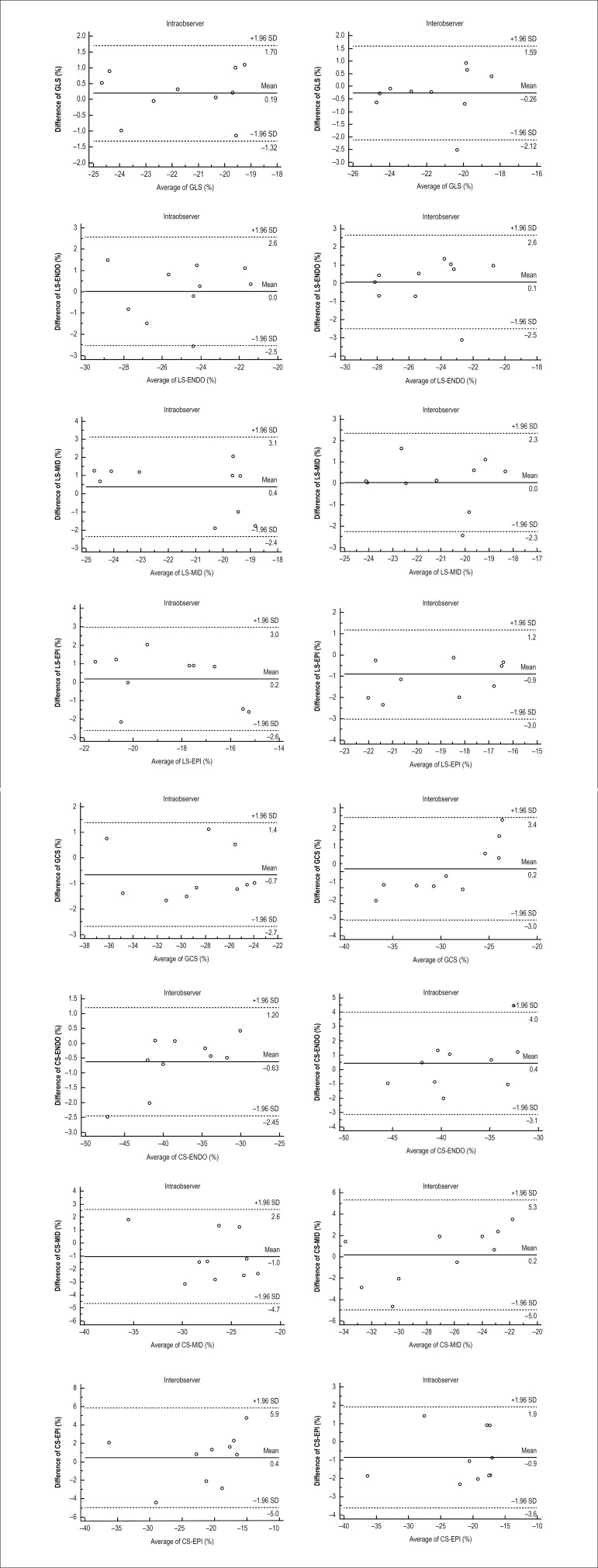
Bland-Altman analysis of inter-, intra-observation reproducibility.

Inter-observer measurement showed ICC = 0.91 for CS-ENDO, 0.83 for CS-MID, 0.91 for CS-EPI, 0.95 for GCS, 0.61 for RS, 0.87 for LS-ENDO, 0.85 for LS-MID, 0.90 for LS-EPI, 0.91 for GLS. Similarly, intra-observer measurement showed ICC = 0.96 for CS-ENDO, 0.89 for CS-MID, 0.97 for CS-EPI, 0.97 for GCS, 0.73 for RS, 0.86 for LS-ENDO, 0.85 for LS-MID, 0.82 for LS-EPI, 0.94 for GLS, indicating satisfactory reproducibility by speckle-tracking-derived multilayer analysis of circumferential and longitudinal strain values. Bland-Altman curves of strain values were shown on Figure 2.

## Discussion

Globally, cancer is diagnosed in 12.7 million people annually, with its incidence projected to increase by 40% in high-income countries from 2008 to 2030.^10^

Anthracyclines are powerful cytotoxic agents, available to treat a wide spectrum of hematologic malignancies and solid tumors. However, life altering cardiac sequelae from anthracyclines remain a problem, with a range of 5-23% of patients developing late-onset heart failure secondary to anthracycline induced-cardiotoxicity.^11^

Reliable, sensitive and non-invasive methods in detecting cardiac function are of vital importance in these patients. The present study demonstrated that subclinical cardiotoxicity existed in long-survivors after receiving anthracycline therapy albeit normal conventional echocardiographic findings, implicating the more sensitive nature of these parameters in monitoring anthracycline cardiotoxicity.

Recently tagged magnetic resonance imaging provided a detailed quantitative analysis of left ventricular transmural differences in myocardial deformation.^12^ Echocardiographic speckle-tracking strain analysis, which is angle-independent, provides a noninvasive method to assess left ventricular mechanics, thus translating clinically relevant aspects of cardiac performance from "bench to bedside". Furthermore, the echocardiographic speckle tracking derived transmural gradients has recently been validated against sonomicrometry crystal in a sheep model.^13^ As proved by previous studies,^14-16^ our observations showed great interobserver and intraobserver agreement, suggesting reasonable reproducibility of the speckle tracking-derived multi-layer strain parameters.

The present study confirmed the presence of transmural and translevel gradient in myocardial circumferential and longitudinal strains, with higher values in the subendocardial myocardial layer and in the apical level in both normal subjects and patients exposed to anthracycline, as is improved by Shi et al.^16^ The difference in amplitude of myocardial contraction between the subendocardial and subepicardial regions was related to the orientation pattern of myocardial fiber in the heart. It has been described that in normal heart, contraction is greater in the subendocardial myocardium layer than in the subepicardial myocardium layer.^17^ However, with greater contraction and higher energy requirements, subendocardial layer was more susceptible to injury, which can be detected by multi-layer speckle tracking strain analysis. Beck et al.^18^ has demonstrated that a multi-layer analysis of myocardial deformation is highly accurate in the differentiation between different degrees of scar transmurality as assessed by MRI. In particular, multi-layer strain analysis provided higher accuracy to discriminate nontransmural versus noninfarction or trasmural versus nontransmural infarction compared with global strain. Altiok et al.^19^ has also found that circumferential endocardial strain analysis allowed accurate distinction between segments with non-transmural infarction vs those with no infarction and between segments with transmural vs non-transmural infarction as defined by late gadolinium enhancement cardiovascular magnetic resonance. In the present study, we adopted a multi-layer strain approach in analyzing layer-specific ventricular deformation and observed the decrease of subendocardial circumferential strain values and transmural circumferential gradient in long-term survivors exposed to anthracycline. It has been proved in animal models of anthracycline cardiotoxicity that severe myocytolysis mainly involved the subendocardium of the ventricle.^20^ Moreover, Perel et al.^21^ observed a regional and diffuse pattern of subendocardial enhancement using cardiac magnetic resonance imaging in patients with anthracycline-induced cardiomyopathy. Hence, the findings in our study of reduction of subendocardial circumferential strain values and transmural circumferential gradient but preserved subepicardial circumferential strain was consistent with the same hypothesis of subendocardial injury induced by anthracycline. Moreover, it has been proved^22^ that in patients with chronic ischemic cardiomyopathy, subendocardial circumferential strain was a powerful predictor of cardiac events and appeared to be a better parameter than LVEF and other strain variables analyzed by echocardiography. Therefore, we believed that further importance may need to be attached to the changes of subendocardial circumferential strains.

We observed that after anthracycline exposure, longitudinal strains of all the three layers decreased significantly. However, transmural longitudinal strain gradient did not show any difference compared to normal group. It is reported that the subendocardium is predominantly composed of longitudinal myocardial fiber. The subendocardial deformation is greatest in the longitudinal direction and verifies the endo-epicardial gradient in normal left ventricles on magnetic resonance imaging.^23,24^ Hence, the longitudinal left ventricular mechanics are predominantly governed by the subendocardial region of the myocardium, which probably accounts for our findings of the reduction of all three layers of longitudinal strain values and the absence of difference in longitudinal transmural gradient.

The lack of difference in radial strain between two groups in our study was perhaps not surprising, which was concordance with some previous studies.^25,26^ It was recently published that peak radial strain differed largely between different software and algorithms, and small changes in width can change large RS differences.^27^ In the present study, the interobserver variation did not show satisfactory reproducibility of RS measurement. Hence, it may suggest that indices of radial deformation are not as sensitive as circumferential and longitudinal strains in detecting subclinical left ventricular dysfunction.

The absence of associations between strain parameters and cumulative anthracycline indicated lack of a safe dose that was free of cardiotoxicity. It has been proved that even children who have received a cumulative doxorubicin dose as low as 45mg/m^2^ have reduced left ventricular mass^28^ and anthracycline damage to all cardiac structures may begin with the first anthracycline dose.^29^

### Limitation

Several limitations to this study warranted comment. This was a cross-sectional study of a relatively small patient cohort, which did not provide information on the value of myocardial deformation parameters in prognostication. Further out-come studies with hard clinical endpoints will be required to determine the clinical significance of our findings. Secondly, although speckle tracking echocardiography allows interrogation of global strain, these parameters are not entirely load dependent and need to be interpreted with caution when an alteration of cardiac status with acute changes in load is anticipated.

## Conclusion

Despite normal left ventricular ejection fraction, subtle abnormalities in myocardial systolic function were present in long-term survivors after anthracycline exposure. It provided the evidence of preferential impairment of subendocardial deformation in long-term survivors after exposure to anthracycline. Multi-layer speckle tracking echocardiography, a potential non-invasive tool for the detection of subclinical anthracycline-induced myocardial abnormalities, might facilitate the longitudinal follow-up of this at-risk patient cohort.
